# Instillation Therapy for the Treatment of Necrotizing Fasciitis: A Case Study

**DOI:** 10.7759/cureus.917

**Published:** 2016-12-07

**Authors:** Douglas F Blazek, Emily Lockridge

**Affiliations:** 1 Cornerstone Surgical Specialists, High Point Regional, UNC Healthcare

**Keywords:** necrotizing fasciitis, wound, instillation, veraflo, npwt, npwti-d, sepsis, diabetes, saline, surgical debridement

## Abstract

A 57-year-old obese female with uncontrolled diabetes mellitus type I presented to the emergency department (ED) with the main complaint of worsening pain and redness in her right groin and inguinal regions. In the ED, a CT scan confirmed the likely presence of necrotizing fasciitis in the right groin and thigh. She was also found to be febrile and septic on admission. She was urgently taken to the OR for extensive debridement then admitted to the ICU for medical stabilization. She was subsequently taken back for serial debridements, and a negative pressure wound therapy with instillation (NPWTi-d) Veraflo device was placed once the wound was successfully debrided down to viable tissue. This is a case study on the wound progression of this patient.

## Introduction

A 57-year-old morbidly obese female with poorly controlled diabetes and sepsis and was found to have necrotizing fasciitis of the right groin and upper inner thigh. She was treated with multiple debridements in the operating room as well as by the ICU-Critical Care for her severe sepsis and diabetes management. She also had severe respiratory distress and required prolonged intubation and tracheostomy prior to discharging to the longterm acute care (LTAC) facility. After her wound had been debrided to viable tissue, a negative pressure wound therapy with instillation (NPWTi-d) Veraflo device was placed and continued for approximately one month until transitioned to negative pressure wound therapy (NPWT) device. Once medically stable, she was discharged to a LTAC facility with the NPWT in place.

## Case presentation

A 57-year-old obese female presented to the emergency department with worsening pain and infection of the right inguinal region, right thigh, and lower abdominal wall. A CT scan confirmed the likely necrotizing fasciitis, and the patient was also found to have poorly controlled diabetes mellitus and sepsis on admission. She was admitted by the Critical Care team and emergently taken to the operating room for incision and drainage, with extensive debridement of her necrotizing fasciitis. The original wound size following this debridement was 60 cm x 30 cm x 15 cm down to the muscle. Her sepsis failed to resolve, and she was, therefore, taken back to the operating room 36 hours later for further debridement down to the muscle with a long segment of visible femoral artery and sciatic nerve exposed. She was taken back to the operating room again four days later for further debridement as she was too unstable during the previous procedures for prolonged anesthesia and blood loss.

**Figure 1:**
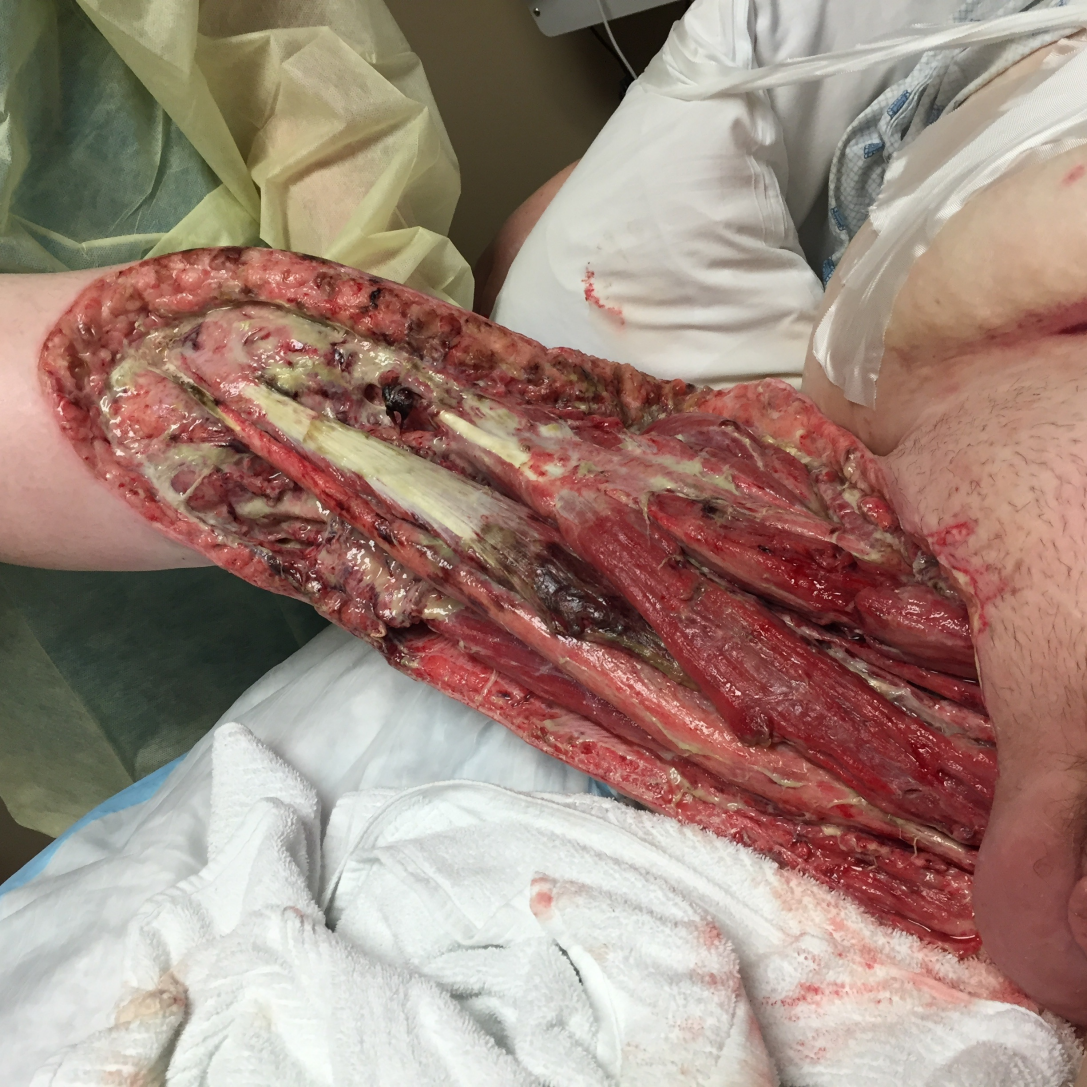
Initial Wound Following Mutliple OR Debridements, Prior to Placement of NPWTi-D Therapy

**Figure 2:**
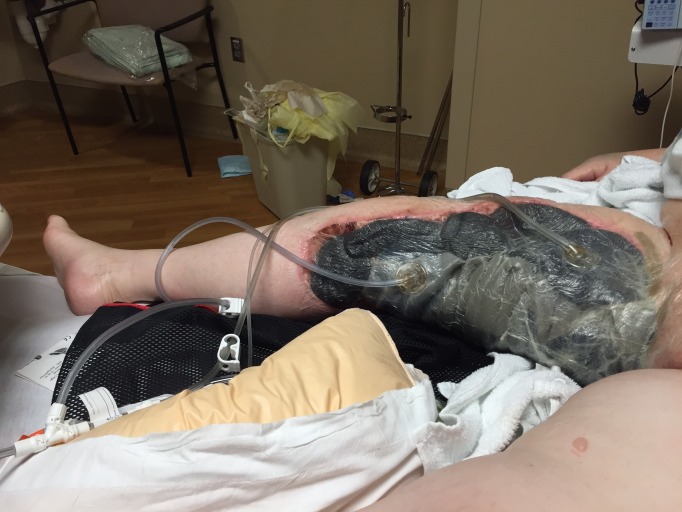
Demonstration of the Extensive Size of Wound Following the First Application of NPWTi-D

Throughout the above-mentioned procedures, her wound was also thoroughly irrigated via pulsavac lavage. After the second debridement due to the severity of her wound, an orthopedic consult was obtained for an opinion on a hip disarticulation; they recommended proceeding with the current wound care and would proceed with disarticulation if she failed with the current therapy. Forty-eight hours following the 3rd OR debridement (Figure 1), an NPWTi-d Veraflo device was placed on the wound with normal saline instillation solution with the following settings: 325 ml normal saline was instilled for a 10 minute soak every three and a half hours under -125 mmHg continuous pressure per the recommendations of the clinical panel [1-4] (Figure 2). Use of normal saline for instillation was chosen as studies have shown that it is as effective as other topical wound cleaning solutions [5-6]. Similar to the use in this patient, multiple clinical trials have shown that NPWTi-D versus NPWT achieves faster wound closure and better skin perfusion when used with -125 mmHg [7-9].

**Figure 3:**
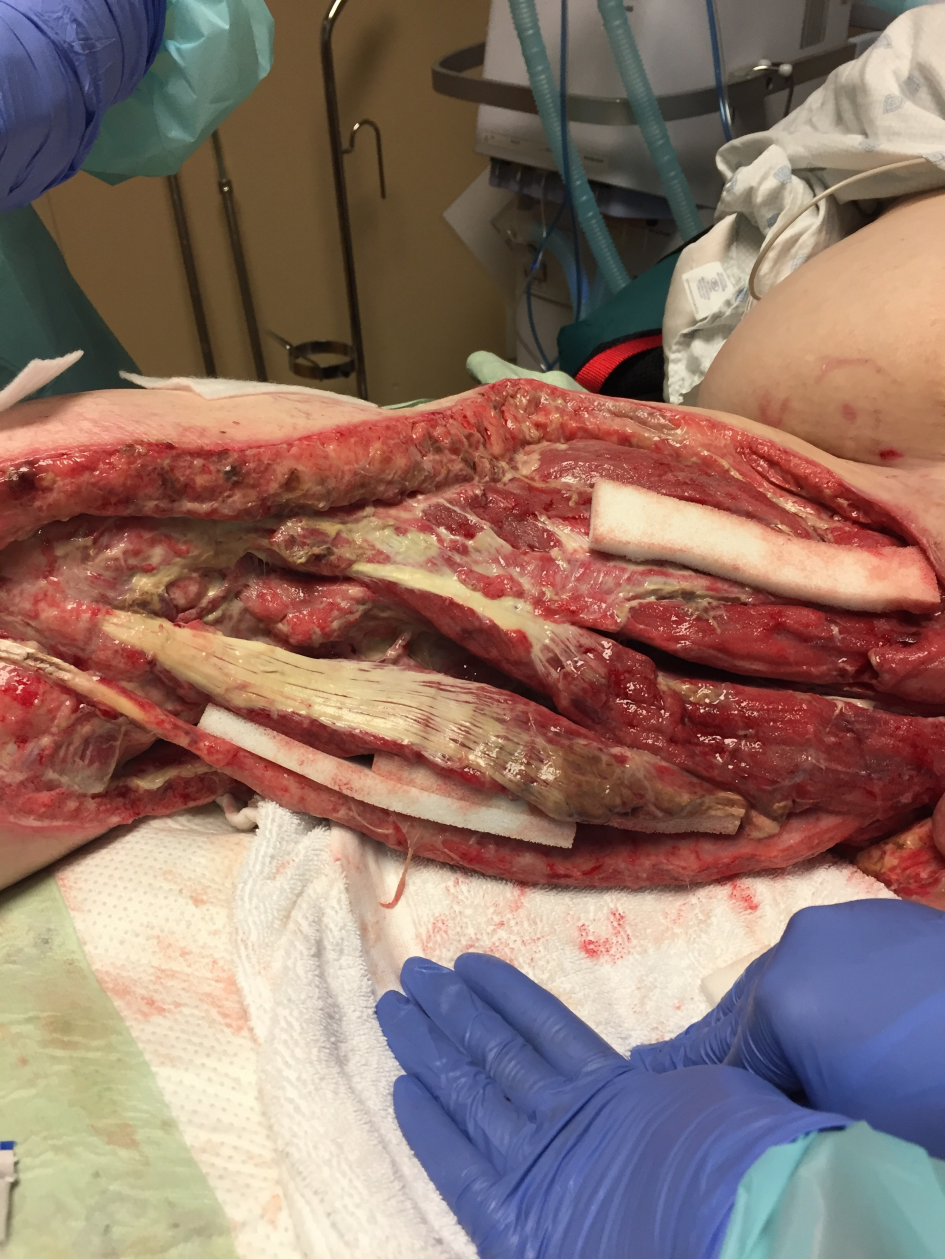
First NPWTi-d Dressing Change White foam still in place which was protecting exposed vasculature and nerve structures.

The dressings were changed on a 3-4 day schedule. After four days of treatment with the NPWTi-d Veraflo, the wound showed improvement with increased granulation tissue and viable skin edges, with granulation over the exposed artery and nerve (Figure 3).

**Figure 4:**
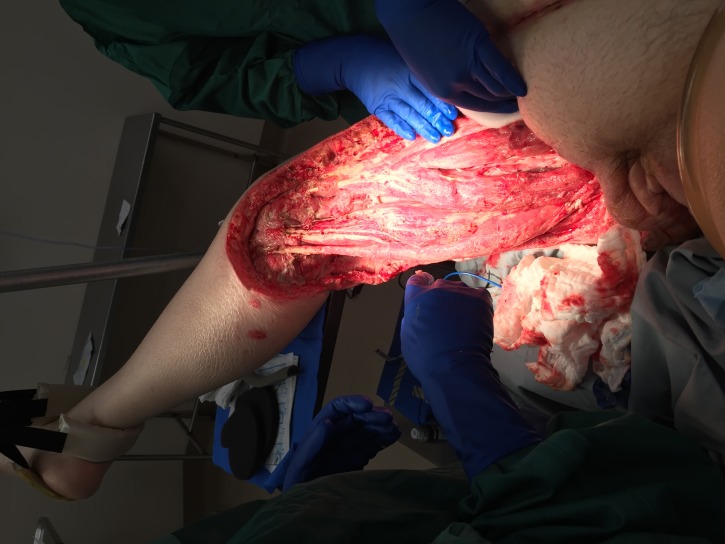
Third Dressing Change Following NPWTi-D Therpay This shows increased granulation over exposed vasculature, nerves, and exposed muscles and tendons.

The patient had a prolonged hospital course. Once she was off sedation and medically stable, due to the size and extent of her wound, she was taken back to the OR three days later for a dressing change, and the overall wound showed great improvement with increased granulation throughout (Figure 4).

**Figure 5:**
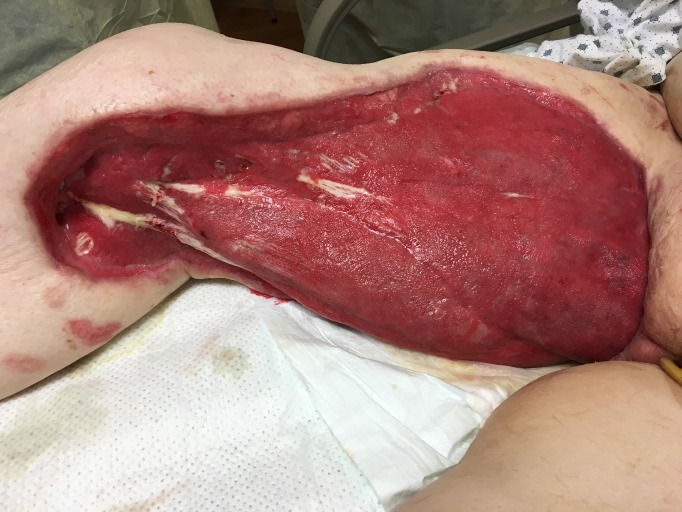
The Wound After the Patient Had Been Transitioned to NPWT, Approximately One Month Following the Initial Placement of NPWTi-D Therapy

NPWTi-d Veraflo therapy was continued with one more subsequent dressing change seven days later and then returned to NPWT -125 mmHg until discharge from the hospital to the LTAC facility. NPWT was continued with dressing changes three times weekly at the outside LTAC facility as discussed for continued therapy for expedited granulation. The patient was then readmitted 20 days later for other medical complications; the wound was evaluated and exhibited almost complete granulation over all exposed muscle, vasculature, and nerve structures--almost superficial to surrounding peri-wound skin (Figure 5).

She was once again discharged to the LTAC facility with NPWT. The patient was then readmitted about 30 days later for VRE pneumonia and expired from this, therefore complete closure of her wound was not able to be achieved. Informed consent was waived, and no reference to the patient's identity was made at any stage during data analysis or in the report.

## Discussion

The following questions should be asked when considering NPWT versus NPWTi-d:

Was the VeraFlo therapy a critical component of this patient's limb salvage?

Did the VeraFlo therapy expedite healing for this patient?

Were the VeraFlo dressing changes easier to manage than standard-of-care dressings?

These are some of the considerations that were taken prior to developing the appropriate treatment plan for this patient. Overall, she had a great response with rapid granulation tissue growth following her NPWTi-d therapy. The authors strongly feel that she could have undergone a muscle flap/split thickness skin grafting to complete her wound closure if she had not unfortunately expired from her other multiple medical comorbidities.

## Conclusions

VeraFlo played a critical role in the limb salvage as orthopedics was consulted for total hip disarticulation for this patient prior to initiation of VeraFlo therapy. Had she not responded so well to the VeraFlo therapy, the hip disarticulation would have ultimately been necessary to save her life. VeraFlo therapy was chosen after reviewing the cited articles, publications, and websites [1-10].

Healing was greatly expedited with the use of VeraFlo therapy in this case. A soft tissue deficit of this magnitude could potentially take years to granulate in and epithelialize. Instead, using VeraFlo, we saw rapid granulation and coverage of exposed nerves, vasculature, and tendons in just weeks. 

The VeraFlo dressings were changed at the bed-side twice per week. Standard-of-care wound dressings for this type of wound are typically done twice daily. This saved significant nursing time and supply costs. VeraFlo therapy also decreased the number of times we needed to sedate the patient to undergo dressing changes. We were ultimately able to cut back on the number of operative debridements, which saved time and expense to the hospital.
